# Evaluation of the Effect Derived from Silybin with Vitamin D and Vitamin E Administration on Clinical, Metabolic, Endothelial Dysfunction, Oxidative Stress Parameters, and Serological Worsening Markers in Nonalcoholic Fatty Liver Disease Patients

**DOI:** 10.1155/2019/8742075

**Published:** 2019-10-15

**Authors:** Alessandro Federico, Marcello Dallio, Mario Masarone, Antonietta Gerarda Gravina, Rosa Di Sarno, Concetta Tuccillo, Valentina Cossiga, Stefania Lama, Paola Stiuso, Filomena Morisco, Marcello Persico, Carmelina Loguercio

**Affiliations:** ^1^Department of Precision Medicine, University of Campania “Luigi Vanvitelli”, Via De Crecchio 7, 80138 Naples, Italy; ^2^Department of Medicine and Surgery, University of Salerno, “Scuola Medica Salernitana” Internal Medicine and Hepatology Unit, Via Allende, 84081 Baronissi, Salerno, Italy; ^3^Department of Clinical Medicine and Surgery, University of Naples “Federico II”, Naples, Italy

## Abstract

Nowadays, the nonalcoholic fatty liver disease represents the main chronic liver disease in the Western countries, and the correct medical therapy remains a big question for the scientific community. The aim of our study was to evaluate the effect derived from the administration for six months of silybin with vitamin D and vitamin E (RealSIL 100D®) on metabolic markers, oxidative stress, endothelial dysfunction, and worsening of disease markers in nonalcoholic fatty liver disease patients. We enrolled 90 consecutive patients with histological diagnosis of nonalcoholic fatty liver disease and 60 patients with diagnosis of reflux disease (not in therapy) as healthy controls. The nonalcoholic fatty liver disease patients were randomized into two groups: treated (60 patients) and not treated (30 patients). We performed a nutritional assessment and evaluated clinical parameters, routine home tests, the homeostatic model assessment of insulin resistance, NAFLD fibrosis score and fibrosis-4, transient elastography and controlled attenuation parameter, thiobarbituric acid reactive substances, tumor necrosis factor *α*, transforming growth factor *β*, interleukin-18 and interleukin-22, matrix metalloproteinase 2, epidermal growth factor receptor, insulin growth factor-II, cluster of differentiation-44, high mobility group box-1, and Endocan. Compared to the healthy controls, the nonalcoholic fatty liver disease patients had statistically significant differences for almost all parameters evaluated at baseline (*p* < 0.05). Six months after the baseline, the proportion of nonalcoholic fatty liver disease patients treated that underwent a statistically significant improvement in metabolic markers, oxidative stress, endothelial dysfunction, and worsening of disease was greater than not treated nonalcoholic fatty liver disease patients (*p* < 0.05). Even more relevant results were obtained for the same parameters by analyzing patients with a concomitant diagnosis of metabolic syndrome (*p* < 0.001). The benefit that derives from the use of RealSIL 100D could derive from the action on more systems able to advance the pathology above all in that subset of patients suffering from concomitant metabolic syndrome.

## 1. Introduction

Nonalcoholic fatty liver disease (NAFLD) represents the major cause of chronic liver disease in the Western countries [1, 2]. Very likely, it will occupy a leading position in the near future among the causes of cirrhosis and hepatocellular carcinoma (HCC) in increasingly younger patients [3]. An important contribution to the progression of the disease from simple steatosis (NAFL) to nonalcoholic steatohepatitis (NASH) is given by the alteration of the oxide-reductive imbalance that involves the cells of various organs and apparatus [4, 5]. However, the mechanisms responsible for this pathological evolution are not yet completely clear. The current attention of clinicians and researchers is oriented towards the possibility of using serum instruments and biomarkers able to provide valuable information on the extent of liver fat accumulation, systemic inflammation, and endothelial dysfunction [6–8]. In fact, NAFLD is closely linked with cardiovascular pathology, representing an independent risk factor for the development of chronic and acute diseases [9, 10]. This linkage is represented, precisely, by the endothelial dysfunction, which, in turn, is caused by the systemic “low-grade inflammation” that is supported both by the alteration of metabolic homeostasis and by the high production of reactive oxygen species in NAFLD patients [11]. In the recent past, scientific research has led to the identification of different serological markers of endothelial dysfunction, of which the most important elevated findings in subjects with NAFLD were high mobility group box 1 (HMGB-1), Endocan, and anti-endothelial cell antibodies (AECAs) [12]. The correct planning of the diagnostic, prognostic, and therapeutic procedures for NAFLD still represents a huge challenge for the scientific community, and in accordance with the clinical practice guidelines, the only therapeutic approach considered effective for this type of patients is nowadays constituted by dietary interventions and exercise. However, studies of our group have shown how the use of 12 months of a therapy with silybin conjugated with phospholipids, and vitamin E, in subjects with histological diagnosis of NASH, is able to improve the NAFLD activity score (NAS), the lipidomic profile, and the serum oxidative state as well as different metabolic parameters in these patients due to the well-known effect of silybin as an antioxidant, antifibrotic, and anti-inflammatory compound [13–15]. Moreover, a vitamin D deficiency in patients with NAFLD and metabolic syndrome exists. Vitamin D is closely related through its receptor to the fibrogenic mechanisms supported by the transforming growth factor-beta (TGF-*β*) in the liver. In particular, it would mediate a reduction in the TGF-*β*-induced fibrotic deposition as it happens during the progression of the disease towards fibrosis and cirrhosis [16, 17]. Therefore, it seems plausible that the administration of substances such as silybin and its association with vitamin D could positively influence the course of the disease by stopping or slowing the evolution of NASH in cirrhosis. For these reasons, the aim of our study was to evaluate the effect derived from the administration for six months of silybin with vitamin D and vitamin E (RealSIL 100D®) on metabolic markers of oxidative stress, endothelial dysfunction, and markers of the disease worsening in NAFLD patients.

## 2. Materials and Methods

### 2.1. Patients

This prospective study is in compliance with ethical guidelines of the Declaration of Helsinki (1975) and has been approved by the ethical committee of the University of Campania “L. Vanvitelli” in Naples (protocol no. 531/2016). 90 consecutive patients with histological diagnosis of NAFLD and 60 patients with diagnosis of reflux disease (not in therapy) as healthy controls followed up at Hepatogastroenterology Divisions of University of Campania “Luigi Vanvitelli” were enrolled between January and October 2017, according to inclusion/exclusion criteria, after signing informed consent. The NAFLD patients were randomized into two groups: treated (60 patients) and not treated (30 patients) (2 : 1 ratio treated vs. not treated). Inclusion criteria were age between 18 and 80 years and diagnosis of NAFLD. Exclusion criteria were use of hepatoprotective drugs; presence of tumors or chronic inflammatory disease such as inflammatory bowel disease, rheumatoid arthritis, systemic lupus erythematosus, or other major systemic diseases; ongoing infections; acute or chronic kidney disease; alcohol or drug abuse history; other causes of chronic liver damage; and psychological/psychiatric problems that could invalidate the informed consent. The definition of the presence/absence of NAFLD and the staging of the disease were diagnosed after the exclusion of other causes of liver diseases, by serological tests and clinical data and by performing a liver biopsy. Medical history, alcohol consumption, drug intake, current drug treatments, smoking habits, and blood pressure were investigated. Weight, height, and waist-to-height ratio (WHtR) were directly measured using standardized devices. The body mass index (BMI) was also calculated by dividing the weight (kg) by the square of height (m). Additional data included routine laboratory tests (blood glucose and insulin, ferritin, C reactive protein (CRP), total cholesterol, low-density lipoprotein (LDL), triglycerides, aspartate (AST) and alanine aminotransferases (ALT), gamma glutamyl transpeptidase (*γ*GT), blood count, and vitamin D) and were obtained by blood peripheral venous samples. The homeostatic model assessment for insulin resistance (HOMA-IR), NAFLD fibrosis score (NFS), and fibrosis-4 (FIB-4) score were calculated in accordance with the specific formulas [18, 19]. It was not prescribed any type of dietary regimen or physical exercise during the study period, both for treated patients and controls. All the analyzed parameters were repeated at baseline (T0) evaluation, after 6 months (T6) of therapy, and at the end of the follow-up period (T12).

### 2.2. Histological Assessment

The absence or presence of NASH was evaluated according to standard histopathologic criteria, and severity of the disease was assessed using the NAS established by Kleiner, as the sum of scores of steatosis, lobular inflammation, and hepatocellular ballooning [20]. NASH was considered as diagnostic for NAS > 5. Fibrosis was scored according to Brunt et al. as stage 0 (none), stage 1 (zone 3 perisinusoidal or portal fibrosis), stage 2 (zone 3 perisinusoidal and periportal fibrosis without bridging), or stage 3 (bridging fibrosis). Hepatocyte ballooning was scored as 0 (none), 1 (few), or 2 (many) [21].

### 2.3. FibroScan and Controlled Attenuation Parameter Evaluation

FibroScan transient elastography (TE) was performed using the FibroScan version 502 (Echosens, Paris, France) with standard probes (M and XL probes) [22]. The XL probe was used when the distance from the skin to the liver capsule exceeded 2.5 cm, as measured by sonographic imaging, and/or when BMI was >30. FibroScan was performed by an expert physician without knowledge regarding the results of the histological picture. The objective was to obtain ten acceptable measurements (defined as a successful LS measurement), with the maximum number of attempts set at 20. The criteria proposed by Boursier et al. were used to consider the measurement “very reliable” (IQR/M ≤ 0.1), “reliable” (0.1 < IQR/M ≤ 0.3 or IQR/M > 0.3 with LS median < 7.1 kPa), or “poorly reliable” (IQR/M > 0.3 with LS median ≥ 7.1 kPa) [23]. Based on controlled attenuation parameter (CAP) scores, we classified the enrolled patients in S0, no steatosis (0%–10% fat; 0–237 dB/m); S1, mild steatosis (11%–33% fat; 238–259 dB/m); S2, moderate steatosis (34%–66% fat; 260–292 dB/m); and S3, severe steatosis (>67% fat; ≥293 dB/m) in accordance with calculation of the attenuation of ultrasonic signals used for TE [24].

### 2.4. Nutritional Assessment

In all subjects, food intake was evaluated by an electronic program (WinFood, Medimatica s.r.l., Martinsicuro, Italy). On the basis of the quantities and qualities of foods consumed, the program elaborates the energy intake and the percentage of macronutrients and micronutrients and calculates the elements in each food. The complete elaboration of intakes shows the list of diet components, the ratio among components and calories, and the subdivision in breakfast, lunch, and dinner. We recorded the food intake of a complete week, including working days and the weekend. Data were compared with the tables of food consumption and recommended dietary intakes of the Italian National Institute of Nutrition and Food Composition Database in Italy [25]. Alcohol use was evaluated with a standardized precodified questionnaire (complete AUDIT test) [26]. The quantity of daily alcohol intake was calculated based on a “drink” that corresponds to about 12 g of pure ethanol [27]. The assessment was repeated at baseline (T0) evaluation, after 6 months (T6), and at the end of the follow-up period (T12).

### 2.5. Thiobarbituric Acid Reactive Substance Assessment

The thiobarbituric acid reactive substance (TBARS) assay was performed using 10 *μ*l of serum. The cromogen TBARS was quantified using a spectrophotometer at a wavelength of 532 nm with 1,1,3,3-tetramethoxyprophane as a standard. The amount of TBARS was expressed as nmol/*μ*g of protein. Presented data are the mean (m) ± standard deviation (SD), resulting from three independent experiments. All the analyzed parameters were repeated at baseline (T0) evaluation, after 6 months (T6), and at the end of the follow-up period (T12).

### 2.6. Worsening Markers and Endothelial Dysfunction Assessment

We determined tumor necrosis factor-alpha (TNF-*α*), TGF-*β*, interleukin- (IL-) 18, IL-22, matrix metalloproteinase 2 (MMP-2), epidermal growth factor receptor (EGFR), insulin growth factor-II (IGF-II), cluster of differentiation- (CD-) 44, HMGB-1, and Endocan concentration after collecting peripheral blood samples and centrifuging them for serum extraction. Sera were tested by the enzyme-linked immunosorbent assay (ELISA), according to the manufacturer's instructions (CLOUD-CLONE CORP. (EGFR and HMGB-1), R&D SYSTEMS a biotechnic brand Quantikine ELISA (IGF-II, IL-18, TGF-*β*, MMP-2, IL-22, and TNF-*α*), and https://MyBioSource.com (TBARS AND Endocan)) [28–30]. All the analyzed parameters were repeated at baseline (T0) evaluation, after 6 months (T6), and at the end of the follow-up period (T12).

### 2.7. Experimental Design

We performed a baseline comparison of analyzed parameters between the NAFLD patient group and healthy control one.

Among enrolled NAFLD patients, 60 were randomized to have oral administration of RealSIL 100D® (303 mg of silybin-phospholipid complex, 10 mg of vitamin D, and 15 mg of vitamin E) twice a day for six months, and 30 to not have any type of intervention. The amount of vitamin E in the drug molecule is not used to obtain a therapeutic effect because it is very low; on the contrary, it was used in order to obtain a molecular stability of the drug in accordance with pharmacoengineers that designed the product.

**Figure 1:**
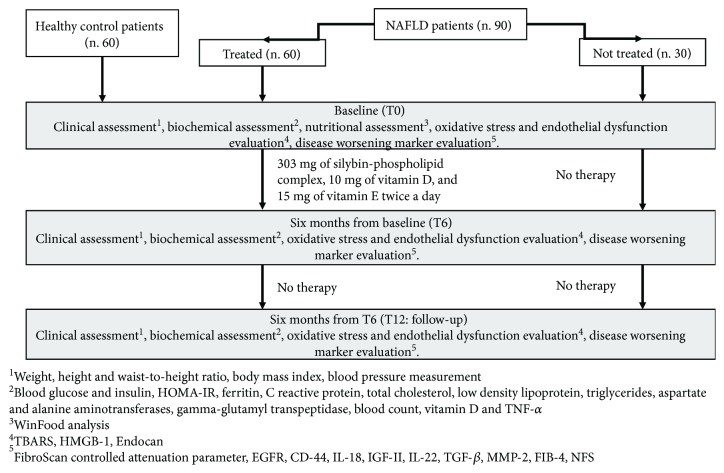
Study design flowchart. NAFLD: nonalcoholic fatty liver disease; HOMA-IR: homeostatic model assessment for insulin resistance; TNF-*α*: tumor necrosis factor-alpha; HMGB-1: high mobility group box 1; TBARS: thiobarbituric acid reactive substances; EGFR: epidermal growth factor receptor; CD: cluster of differentiation; IL: interleukin; IGF: insulin growth factor; TGF-*β*: transforming growth factor-beta; MMP-2: metalloproteinase 2; FIB-4: fibrosis 4 index; NFS: NAFLD fibrosis score.

Then, all patients were followed up for another 6 months without therapy. At the baseline, we performed a nutritional assessment. During the period of the study, patients were on free diet on the basis of dietary habits prior to the enrollment, and any type of physical exercise was prescribed during the study period. Moreover, we performed at the baseline (T0), at the end of the treatment period (T6), and after six months of follow-up (T12) the clinical, biochemical, liver fibrosis/steatosis, oxidative stress, and endothelial dysfunction assessment (Figure 1). In the evaluation of all the studied parameters, we considered as “improved” the normalization of the specific variable under the upper limit of the normality range level.

### 2.8. Statistical Analysis

The number of patients (90; 60 in the interventional arm and 30 in the observational one) was calculated on the basis of one of the endpoints of the study, namely, the reduction in CAP. A supposed significant reduction was calculated on the basis of the CAP validation studies that showed how a difference of about 20 dB/m identified a difference between steatosis classes with a good diagnostic performance (S1: 220-240 dB/m, S2: 230-260 dB/m, and S3: 260-300 in the various studies) [31]. On the basis of those data, we calculated that the number of patients needed to measure a statistically significant difference of about 20 dB/m before and after treatment, with a power of 90% and an alpha error of 0.01 in a two-tailed test for paired samples in repeated measures (before and after treatment), was 27 patients per arm (calculation performed with STATA v14 package for Mac: Power And Sample size calculation for means, repeated measures-StataCorp. 2015. Stata Statistical Software: Release 14. College Station, TX: StataCorp LP). Subsequently, to improve the possibilities of collecting significant differences also in the laboratory parameters, it was decided to enroll 30 patients in the observation arm and to double the number (60) in the interventional arm. Parametric and nonparametric tests were performed to compare the continuous variables when appropriate. In particular, the Student *t*-test and Mann–Whitney *U* test were performed to compare continuous variables; chi-square with Yates correction or the Fisher-exact test was performed to compare categorical variables. Data were reported as the mean ± standard deviation for continuous variables with a normal distribution and as the median and interval for those with “nonnormal” distribution. To assess if continuous variables were normally or not normally distributed, we preliminarily performed a Kolmogorov-Smirnov “goodness of fit” test for normality. Statistical significance was defined when “*p* < 0.05” in a “two-tailed” test with a 95% confidence interval. Statistical analyses were performed using the Statistical Program for Social Sciences (SPSS®) 20.0 for Macintosh® (SPSS Inc., Chicago, Illinois, USA).

## 3. Results and Discussion

### 3.1. Results

The general characteristics of the enrolled patients are shown in Table 1.

**Figure 2:**
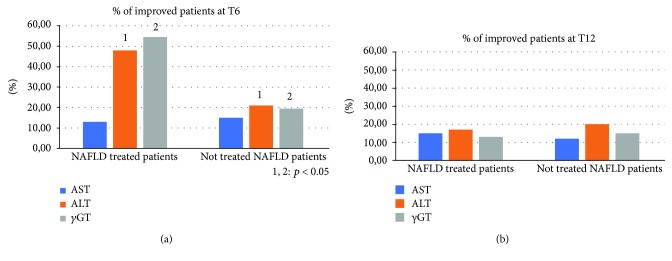
Comparison between the two NAFLD group patients with improvement of aspartate/alanine aminotransferase and gamma glutamyl transpeptidase. NAFLD: nonalcoholic fatty liver disease; AST: aspartate aminotransferase; ALT: alanine aminotransferase; *γ*GT: gamma glutamyl transpeptidase.

**Figure 3:**
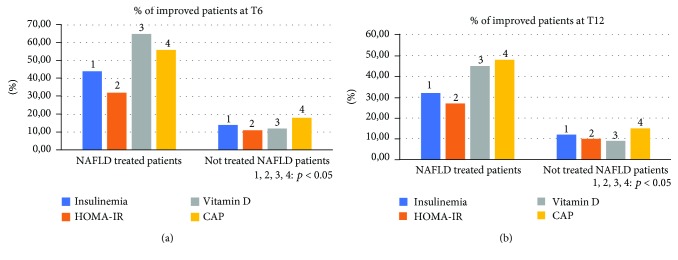
Comparison between the two NAFLD group patients with improvement of insulinemia, the homeostatic model assessment for insulin resistance, vitamin D, and controlled attenuation parameter. NAFLD: nonalcoholic fatty liver disease; HOMA-IR: homeostatic model assessment for insulin resistance; CAP: controlled attenuation parameter.

**Figure 4:**
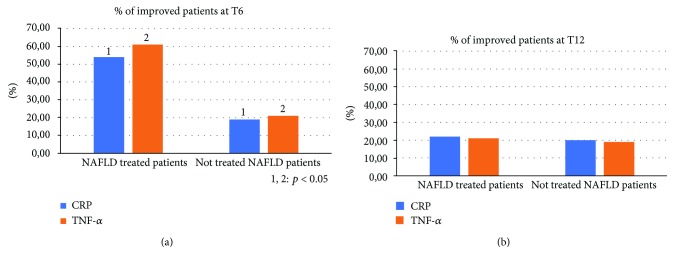
Comparison between the two NAFLD group patients with improvement of C reactive protein and tumor necrosis factor-alpha. NAFLD: nonalcoholic fatty liver disease; CRP: C reactive protein; TNF-*α*: tumor necrosis factor-alpha.

**Figure 5:**
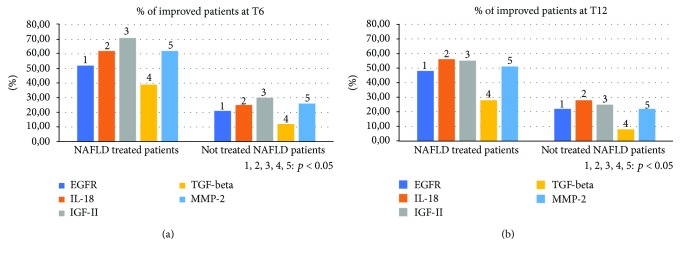
Comparison between the two NAFLD group patients with improvement of epidermal growth factor receptor, interleukin-18, insulin growth factor-II, transforming growth factor-beta, and matrix metalloproteinase-2. NAFLD: nonalcoholic fatty liver disease; EGFR: epidermal growth factor receptor; IL-18: interleukin-18; IGF-II: insulin growth factor-II; TGF-beta: transforming growth factor-beta; MMP-2: matrix metalloproteinase-2.

**Figure 6:**
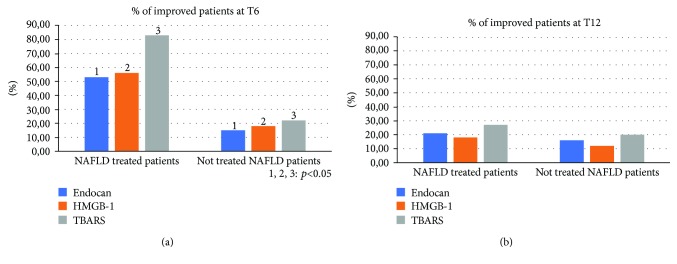
Comparison between the two NAFLD group patients with improvement of Endocan, high mobility group box-1, and thiobarbituric acid reactive substances. NAFLD: nonalcoholic fatty liver disease; HGMB-1: high mobility group box-1; TBARS: thiobarbituric acid reactive substances.

Compared to the population of healthy control patients, NAFLD patients had statistically significant differences (*p* < 0.05) for almost all the parameters evaluated at the baseline: BMI, body weight, AST, ALT, insulin, HOMA-IR, total cholesterol, triglycerides, vitamin D, CRP, TNF-*α*, EGFR, CD-44, IL-18, IGF-II, IL-22, TGF-*β*, MMP-2, Endocan, HMGB-1, and TBARS (Table 1). No statistically significant differences were found regarding the daily caloric intake and the type of daily calories between the two groups of NAFLD patients (treated vs. not treated) (Table 2). Furthermore, the repetition of the nutritional assessment at the three observation times envisaged by the study (T0, T6, and T12) did not reveal significant changes in dietary habits for both the groups: treated and the not treated NAFLD. Regarding the clinical parameters evaluated (BMI, WHtR, weight, and blood pressure), no significant differences were found between the two NAFLD groups and, within each group, between the three observation study times. Six months after the baseline, the proportion of treated NAFLD patients who experienced a statistically significant improvement in ALT and *γ*GT was greater compared to not treated NAFLD patients (*p* = 0.046 and *p* = 0.032, respectively). On the other hand, there was no significant change in AST in the two groups of patients at six months from the baseline (T6) (*p* = 0.073) (Figure 2(a)). At the end of the follow-up period (T12), the proportion of NAFLD patients treated that showed a significant improvement of ALT and *γ*GT was significantly reduced, becoming similar to that of the not treated (*p* = 0.143; *p* = 0.091). The AST did not undergo significant changes at the baseline, T6, and T12 (Figure 2(b)). With regard to metabolic parameters, the proportion of patients treated that showed a statistically significant improvement in insulin, HOMA-IR, vitamin D, and degree of steatosis assessed by CAP at six months from the baseline was greater than that in not treated patients, even if for this last parameter a complete normalization was not observed after six months of treatment (*p* = 0.032, *p* = 0.044, *p* = 0.038, and *p* = 0.042, respectively) (Figure 3(a)). This difference remained statistically significant at T12 (*p* = 0.041, *p* = 0.043, *p* = 0.033, and *p* = 0.048, respectively) (Figure 3(b)). With regard to glycaemia, total cholesterol, triglycerides, and LDL, there were no significant changes at the three observation times for both NAFLD groups. Among the parameters of systemic inflammation, the proportion of improved patients at T6 compared to the baseline was greater in the treated group compared to the not treated regarding CRP and TNF-*α* (*p* = 0.03 and *p* = 0.037, respectively) (Figure 4(a)). These parameters in the treated group became similar to not treated patients at T12 (*p* = 0.112 and *p* = 0.657, respectively) (Figure 4(b)). There were no significant changes in ferritin at the three observation times for both NAFLD groups. Among the markers of worsening/disease progression, the proportion of treated patients which presented a significant improvement at T6 compared to the baseline of EGFR, IL-18, IGF-II, TGF-*β*, and MMP-2 was greater than the not treated patients (*p* = 0.044, *p* = 0.041, *p* = 0.032, *p* = 0.033, and *p* = 0.021, respectively) (Figure 5(a)). At T12, this data was confirmed (*p* = 0.046, *p* = 0.039, *p* = 0.042, *p* = 0.043, and *p* = 0.036, respectively) (Figure 5(b)). On the contrary, no significant changes were found at the three observation times for CD-44, IL-22, FIB-4, NFS, and stiffness in the two groups of patients. Finally, the proportion of patients who showed at T6 compared to the baseline a significant improvement in Endocan, HMGB-1, and TBARS was greater in the group of patients treated compared to NAFLD controls (*p* = 0.045, *p* = 0.043, and *p* = 0.031) (Figure 6(a)). At T12, the proportion of patients with improvement of these parameters returned to be similar in the two NAFLD study groups (*p* = 0.14, *p* = 0.082, and *p* = 0.091) (Figure 6(b)).

**Figure 7:**
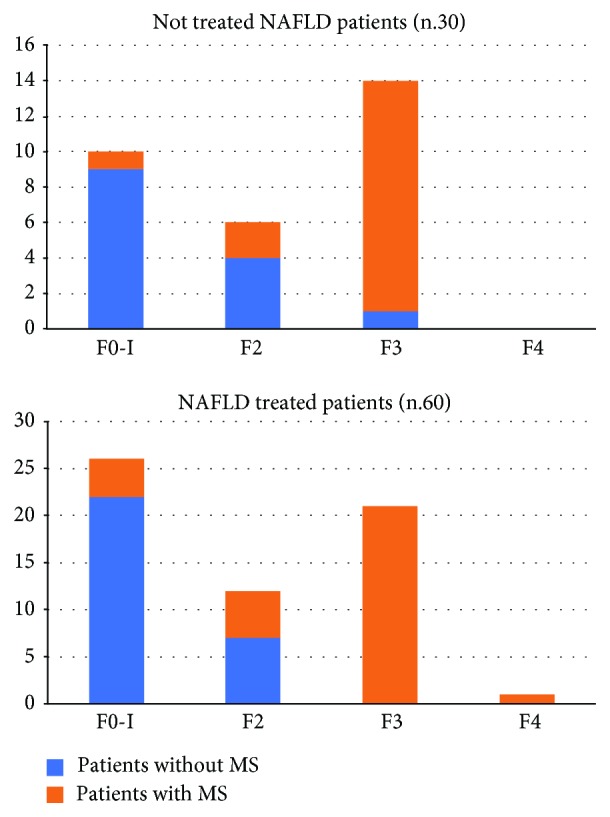
Fibrosis degree distribution in the two NAFLD groups with or without metabolic syndrome. NAFLD: nonalcoholic fatty liver disease; MS: metabolic syndrome.

**Figure 8:**
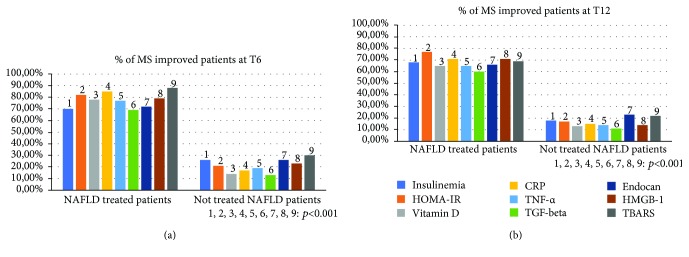
Comparison between the two NAFLD group patients with metabolic syndrome which presented an improvement of insulinemia, the homeostatic model assessment for insulin resistance, vitamin D, C reactive protein, tumor necrosis factor-alpha, transforming growth factor-beta, Endocan, high mobility group box-1, and thiobarbituric acid reactive substances. NAFLD: nonalcoholic fatty liver disease; MS: metabolic syndrome; HOMA-IR: homeostatic model assessment for insulin resistance; CRP: C reactive protein; TNF-*α*: tumor necrosis factor-alpha; TGF-beta: transforming growth factor-beta; HGMB-1: high mobility group box-1; TBARS: thiobarbituric acid reactive substances.

Sixteen out of 30 (53.3%) not treated NAFLD patients showed the criteria for the diagnosis of metabolic syndrome (MS). Among these, 14/16 (87.5%) presented a histological picture of NASH, with values of NAFLD activity score (NAS) ≥ 6. None of the patients enrolled in this group was classified as F4 in accordance with Metavir staging. Furthermore, 31/60 (51.6%) NAFLD treated patients over the histologically diagnosed NAFLD presented the criteria for the diagnosis of MS. Among them, 30/31 (97%) presented a histological picture of NASH, with NAS values ≥ 6. Regarding the histological staging of fibrosis, only one of the enrolled patients was classified as F4 in accordance with Metavir staging. In the NASH population with MS, higher fibrosis stages have been observed compared to patients with simple steatosis (Figure 7). Analyzing the population subset with MS (47/90 NAFLD study population patients, 16/30 not treated patients; 31/60 treated patients), we observed higher proportions of patients improved at T6 in comparison to the baseline in the group of treated patients compared to the not treated group for the following parameters: insulinemia, HOMA-IR, vitamin D, CRP, TNF-*α*, TGF-*β*, Endocan, HMGB-1, and TBARS (*p* < 0.001) (Figure 8(a)). This observation remained statistically significant even at T12 (*p* < 0.001) (Figure 8(b)).

## 4. Discussion

NAFLD has been the emerging cause of chronic liver disease for several years and will be responsible for the onset of new cases of HCC in the near future, eventually becoming the first indication for liver transplantation [32, 33]. NAFLD often fits into a pathological context that is much more complex than that of other liver diseases. In fact, it is frequently part of a pathological condition involving multiple systems identified with the term MS [34, 35]. This harmful union, whose pathogenesis is still not completely clear, is responsible for two very important consequences. While on the one hand speaking about a “metabolically ill” patient may mean having to consider in the prognosis even extrahepatic diseases such as cardiovascular diseases, on the other, the lack of knowledge of many of the mechanisms responsible for the onset of such conditions and the complex interaction between them does not allow, at present, to design a suitable therapy capable of impacting decisively on the natural history of the disease, stopping its course [36, 37]. The worrying clinical scenario that is recalled when thinking about NAFLD has led in recent years to a great scientific fervor in search of the most appropriate treatment for this type of patients [38]. Several therapeutic attempts have been made in the recent past without obtaining significant results, sometimes even controversial. This is the case, for example, of long-term treatment with insulin-sensitizers, vitamin E, pioglitazone, or their association [38–41].

Several studies reported the role of the intestinal microbiota as a very important factor involved in the genesis of the inflammatory cascades correlated with the NAFLD and the MS, but there are not yet sufficient scientific evidences to support the transferability of the results highlighted by some studies on this topic in daily clinical practice [42]. As if this was not enough, even if many molecules are currently being studied for NAFLD long-term therapy, the majority of the ongoing trials will still require several years before allowing the approval of prescription drugs in this setting [43]. Consequently, the only universally accepted therapy available in this medical picture is represented by diet and physical exercise which have an extraordinary impact on the progression of NAFLD, also slowing down the onset of hepatic and extrahepatic complications [44, 45]. The beneficial effects of herbal product, in particular silybin, on the liver and systemic metabolism have long been studied by many research groups. Silybin antioxidant, insulin-sensitizing, and hepatoprotective capacity, in addition to its high safety profile for long-term administration, led it to represent a “greedy therapeutic opportunity” in the context of metabolic diseases, especially NAFLD [46]. In a multicenter, phase III, double-blind clinical trial, our group had already highlighted how a 12-month treatment with silybin phytosome complex (silybin plus phospholipids) coformulated with vitamin E was able to induce improvement in liver enzymes blood levels, insulin resistance, and liver histology [14]. In this study, we have shown how our population of NAFLD subjects showed a statistically significant difference at the baseline compared to a population of hepatologically healthy subjects (with only reflux disease not being treated) for several of the parameters evaluated. Specifically, NAFLD patients demonstrated greater BMI, body weight, insulin, HOMA-IR, total cholesterol, triglycerides, CRP, TNF-*α*, EGFR, CD-44, IL-18, IGF-II, IL-22, TGF-*β*, MMP-2, Endocan, HMGB-1, and TBARS, with the evidence, moreover, of lower average levels of vitamin D. This pathological picture fully reflects the data present in the scientific literature showing how patients affected by NAFLD are more exposed to oxidative stress, systemic inflammation, and endothelial dysfunction and have a higher blood level of inflammatory cytokines and fibrosis markers compared to healthy subjects, thus being more predisposed to all the pathologies supported by these harmful factors [47–50]. Taking therefore in analysis the NAFLD population randomized in two arms: treated (no. 60) and not treated (nos. 30), there were not found statistically significant differences regarding the daily caloric intake and the type of calories taken daily. Furthermore, food habits of all the enrolled subjects did not change during the study period as we did not recommend patients to adopt a different lifestyle than they had before enrollment to not invalidate the analysis of the results of our study. Furthermore, we did not find any significant variation in the clinical parameters evaluated during the study between the two NAFLD groups. The group of NAFLD treated patients with respect to the not treated NAFLD patients showed a statistically greater proportion of subjects with normalization of the following parameters at six months: ALT, *γ*GT, insulinemia, HOMA-IR, and vitamin D, with an associated reduction in CAP. A similar observation was found for CRP, TNF-*α*, EGFR, IL-18, IGF-II, TGF-*β*, MMP-2, Endocan, HMGB-1, and TBARS. At T12, we observed a clear reduction in the proportion of improved NAFLD treated patients compared to not treated NAFLD patients for ALT, *γ*GT, CRP, TNF-*α*, Endocan, HMGB-1, and TBARS. Otherwise, the advantage gained during the six months of treatment was maintained at T12 for insulinemia, HOMA-IR, vitamin D, CAP, EGFR, IL-18, IGF-II, TGF-*β*, and MMP-2. These evidences show how the use of RealSIL 100D for six months was able, due to the known antioxidant, anti-inflammatory, and insulin-sensitizing effects of silybin, to slow down the pathological process by acting on multiple therapeutic targets. This could mean that RealSIL 100D would be able not only to slow down the progression of liver damage but also to improve the sensitivity of peripheral tissues to insulin by inhibiting the formation of free radicals and acting as scavengers for the latter, reducing lipid peroxidation and membrane permeability [51, 52]. Furthermore, it would be able to act on hepatic stellate cells by inhibiting the extracellular signal-related kinase (ERK) activity, MAP/ERK kinase (MEK), and Raf phosphorylation, reducing the migration of leukocytes to the site of inflammation and reducing TGF-*β*-induced synthesis of type I procollagen as well as MMP-2 secretion [53]. These biological activities are responsible for controlling the inflammatory cascade, the deposition of fat accumulation in the hepatocytes, and the reduction of hepatic and extrahepatic deposition of fibrotic tissue [47–50]. An interesting point is represented by the fact that the improvement of the markers of disease worsening assessed is maintained well beyond the end of the treatment period. How much this reduction results in effective fibrolysis and/or lipolysis or reduction in the risk of HCC development is not known, and further studies are needed. However, it is reasonable to hypothesize how the reduction of the factors that trigger and sustain fibrogenesis can be associated in the long term with a lower deposition of collagen fibers in the organic parenchyma and in the vascular walls. On the contrary, in our clinical setting, the anti-inflammatory and antioxidant effect exerted by silybin seems much more closely connected to its daily administration, running out in a period of time less than six months once the treatment has been interrupted, which would involve the need for a longer administration. Also of great importance is the effect that silybin exerts on the endothelial homeostasis [12]. It now seems scientifically proven, in fact, that the patient suffering from NAFLD, especially in the case of concomitant presence of MS, is a patient burdened by a high cardiovascular risk [9]. The proportion of treated NAFLD patients that showed a marked improvement of Endocan and HMGB-1 compared to not treated NAFLD patients was significantly greater. However, this observation was canceled at the end of the follow-up period, demonstrating, once again, a clear dependence on the administration of the drug. This condition entails the need in the prognostic evaluation of the patient to refer to more medical specialties that should work in concert in order to act on more points in the contrast of the pathology. In this regard, in our clinical setting, the proportion of NAFLD patients treated with concomitant MS that improved in the parameters evaluated with respect to the not treated NAFLD patients with concomitant MS was clearly greater, with levels of statistical significance higher than those obtained from the analysis of the results on the general NAFLD population. This last observation would lead to the hypothesis that the actual benefit that results from a therapy with RealSIL 100D is higher in patients affected by concomitant MS in which NAFLD would be able to progress more rapidly towards more advanced stages of disease in the absence of an appropriate therapeutic intervention.

## 5. Conclusions

In the era of modern hepatology, NAFLD represents the most frequent cause of chronic liver disease in the Western world. The need to understand the pathogenetic mechanisms that support the disease and to connect it with other organs and systems is inextricably linked to the possibility of developing an appropriate therapeutic plan, able to slow down or stop the course of the disease. The close connection between the cardiovascular system and the liver is certainly a key to direct the development of new therapeutic regimes capable of providing concrete answers to the questions of clinicians, as well as the main reason why the management of this pathological condition should be approached with a multidisciplinary perspective. In this picture, the use of treatment strategies that demonstrate to have more therapeutic targets could represent an important turning point for the correct clinical management of NAFLD, especially in a subset at greater risk of pathological evolution.

## Figures and Tables

**Table 1:** General characteristics of the enrolled patients (mean ± SD).

Variable	Healthy controls (no. 60)	NAFLD population (no. 90)	NAFLD treated patients (no. 60)	Not treated NAFLD patients (no. 30)
Age (y)	47 ± 15	54 ± 11	51 ± 6	47 ± 10
Sex (M/F)	30/30	48/42	29/31	19/11
Weight (kg)	71 ± 10.6	88.98 ± 15.08	80.61 ± 13.82	82.07 ± 11.54
BMI (kg/m^2^)	26.4 ± 3.9	32.38 ± 4.56	28.92 ± 6.65	29.43 ± 4.65
WHtR	0.81 ± 0.11	0.94 ± 0.06	0.91 ± 0.16	0.93 ± 0.08
Systolic blood pressure (mmHg)	120 ± 13	141 ± 14	138 ± 16	140 ± 9
Controlled attenuation parameters (dB/m)	168.25 ± 52.05	281.75 ± 60.05	282.65 ± 52.53	279.63 ± 58.76
AST (IU/l)	28 ± 11	44 ± 18	44 ± 9	43 ± 10
ALT (IU/l)	29 ± 14	47 ± 19	45 ± 14	48 ± 16
*γ*GT (IU/l)	30 ± 13	36 ± 5	31 ± 17	39 ± 5
FPG (mg/dl)	72 ± 12	89 ± 26	87 ± 13	90 ± 11
Insulinemia (*μ*U/ml)	12 ± 4	25 ± 8	24 ± 5	26 ± 3
HOMA-IR	0.9 ± 0.2	2.4 ± 0.6	2.5 ± 0.1	2.3 ± 0.4
TCH (mg/dl)	112 ± 30	142 ± 17	138 ± 27	146 ± 22
TG (mg/dl)	87 ± 13	87 ± 13	87 ± 13	87 ± 13
LDL (mg/dl)	89 ± 11	92 ± 9	95 ± 11	90 ± 16
Vitamin D (ng/ml)	88 ± 19	26 ± 15	23 ± 12	27 ± 9
CRP (*μ*g/mg)	0.23 ± 0.02	1.89 ± 0.25	1.76 ± 0.32	2.01 ± 0.06
Ferritin (*μ*g/l)	143 ± 34	156 ± 25	150 ± 32	158 ± 41
TNF-*α* (pg/ml)	12.7 ± 2.2	65.7 ± 22.6	63.5 ± 1.2	68.2 ± 1.8
EGFR (ng/ml)	10.6 ± 5	28.9 ± 2	27.3 ± 4.2	29.8 ± 5.3
CD-44 (ng/ml)	6.1 ± 1.8	12.9 ± 0.7	10.8 ± 0.9	13.3 ± 1
IL-18 (pg/ml)	70.1 ± 38.9	165.6 ± 26.7	159.8 ± 18.9	167.2 ± 22.8
IGF-II (pg/ml)	192.1 ± 43.6	265.9 ± 44.7	261.6 ± 34.6	266.9 ± 32.2
IL-22 (pg/ml)	19.3 ± 6.2	28.8 ± 7.6	29.3 ± 5.2	26.6 ± 4.8
TGF-*β* (pg/ml)	112.3 ± 42.3	188.8 ± 32.1	192.4 ± 41.1	184.2 ± 37.6
MMP-2 (ng/ml)	15.1 ± 3.7	27.4 ± 2.8	29.3 ± 2.3	25.7 ± 3.9
FIB-4	1.17 ± 0.21	1.27 ± 0.34	1.31 ± 0.18	1.24 ± 0.21
NFS	‐1.145 ± 0.02	‐1.149 ± 0.06	‐1.152 ± 0.05	‐1.138 ± 0.04
Stiffness (kPa)	3.4 ± 1.2	5.5 ± 2.6	5.9 ± 0.8	5.1 ± 1.1
Endocan (pg/ml)	372.8 ± 189.3	564.9 ± 196.7	572.3 ± 144.8	541.1 ± 178.9
HMGB-1 (pg/ml)	832.7 ± 242.2	1756.8 ± 212.8	1766.7 ± 282.8	1741.4 ± 142.6
TBARS (nmol/*μ*g)	50.6 ± 24	194.6 ± 32	199.7 ± 27	191.6 ± 14

NAFLD: nonalcoholic fatty liver disease; BMI: body mass index; WHtR: waist-to-height ratio; AST: aspartate aminotransferase; ALT: alanine aminotransferase; *γ*GT: gamma glutamyl transpeptidase; FPG: fasting plasma glucose; HOMA-IR: homeostatic model assessment for insulin resistance; TCH: total cholesterol; TG: triglycerides; LDL: low-density lipoprotein; CRP: C reactive protein; TNF-*α*: tumor necrosis factor-alpha; EGFR: epidermal growth factor receptor; CD-44: cluster of differentiation 44; IL-18: interleukin-18; IGF-II: insulin growth factor-II; IL-22: interleukin-22; TGF-*β*: transforming growth factor-beta; MMP-2: metalloproteinase-2; HMGB-1: high mobility group box-1; TBARS: thiobarbituric acid reactive substances.

**Table 2:** Nutritional assessment of the enrolled patients (mean ± SD).

Variable	Healthy controls (no. 60)	NAFLD population (no. 90)	NAFLD treated patients (no. 60)	Not treated NAFLD patients (no. 30)
Daily intake (kcal)	2116.5 ± 679.5	2746 ± 164	2667 ± 174	2765 ± 144
Total daily proteins (% of total energy intake)	23 ± 11.5	26.3 ± 2.9	24.3 ± 2.3	23.3 ± 5.4
Soluble carbohydrates (g/day)	89.5 ± 26.5	98.9 ± 13.4	89.3 ± 12.4	88.8 ± 16.5
Saturated fatty acids (% of total energy intake)	8 ± 3.95	10.5 ± 1.9	12.3 ± 1.2	12.4 ± 2.4
Monounsaturated fatty acids (% of total energy intake)	4.55 ± 1.3	14.5 ± 4.6	12.8 ± 3.5	13.2 ± 3.8
Polyunsaturated fatty acids (% of total energy intake)	22.5 ± 6.5	7.1 ± 4.2	7.5 ± 4.3	6.5 ± 4.1
Folic acid (*μ*g per day)	342 ± 101.5	328.6 ± 134.5	332.6 ± 134.5	316.6 ± 134.5
Vitamin A (*μ*g per day)	723 ± 199.5	881.4 ± 344.5	798.4 ± 284.3	898.5 ± 351.2
Vitamin C (*μ*g per day)	118 ± 50.5	145.7 ± 49	145.7 ± 49	145.7 ± 42
Thiamine (*μ*g per day)	0.9 ± 0.4	1.6 ± 0.7	1.3 ± 0.2	1.8 ± 0.5
Riboflavin (*μ*g per day)	3.5 ± 2.5	2.6 ± 1.3	2.6 ± 1.3	2.6 ± 1.3
Vitamin B6 (*μ*g per day)	2 ± 0.5	1.2 ± 0.2	0.9 ± 0.5	1.3 ± 0.1

NAFLD: nonalcoholic fatty liver disease.

## Data Availability

The numerical data used to support the findings of this study are included within the article.
